# Indoor concentrations of VOCs in beauty salons; association with cosmetic practices and health risk assessment

**DOI:** 10.1186/s12995-018-0213-x

**Published:** 2018-09-27

**Authors:** Mostafa Hadei, Philip K Hopke, Abbas Shahsavani, Mahbobeh Moradi, Maryam Yarahmadi, Baharan Emam, Noushin Rastkari

**Affiliations:** 1 Research Center for Environmental Determinants of Health (RCEDH), Kermanshah University of Medical Sciences, Kermanshah, Iran; 2 Department of Public Health Sciences, University of Rochester School of Medicine and Dentistry, Rochester, NY 14642 USA; 3 Center for Air Resources Engineering and Science, Clarkson University, Potsdam, NY 13699 USA; 4 Environmental and Occupational Hazards Control Research Center, Shahid Beheshti University of Medical Sciences, Tehran, Iran; 5 Department of Environmental Health Engineering, School of Public Health and Safety, Shahid Beheshti University of Medical Sciences, Tehran, Iran; 6 Environmental and Occupational Health Center, Ministry of Health and Medical Education, Tehran, Iran; 7 Center for Air Pollution Research (CAPR), Institute for Environmental Research (IER), Tehran University of Medical Sciences, Tehran, Iran

**Keywords:** Benzene, Formaldehyde, Toluene, Xylene, Air pollution, Hairdressing

## Abstract

**Background:**

The use of cosmetic products in beauty salons emits numerous kinds of toxic air pollutants. The objectives of this study were to measure the concentrations of benzene, toluene, ethylbenzene, xylene, formaldehyde, and acetaldehyde in 20 large beauty salons in Tehran and relate the observed concentrations to environmental and occupational characteristics of the salons.

**Methods:**

Samples were collected from inside and outside air of 20 selected salons located in different areas of the city. Several additional parameters were recorded during the sampling process including surface area, number of active employees, type of ventilation, type of ongoing treatments, temperature, humidity. Deterministic and stochastic health risk assessment of the compounds were performed.

**Results:**

Indoor concentrations of each pollutant were significantly higher than its outdoor concentrations. Health risk assessment showed that benzene, formaldehyde and acetaldehyde represent a possible cancer risk in the beauty salons. In addition, toluene, ethylbenzene, and xylene had negligible non-carcinogenic risks. Ventilation with air purifier, and fan with open window were more effective than using just a fan. Concentrations of benzene and toluene were affected by the number of hair dying treatments. The concentration of xylene was affected by the number of hair styling. The concentration of formaldehyde was affected by the number of hair styling and number of nail treatments.

**Conclusion:**

With improved ventilation and requirements for reformulated cosmetic, concentrations of toxic air pollutants in beauty salons could be reduced.

**Electronic supplementary material:**

The online version of this article (10.1186/s12995-018-0213-x) contains supplementary material, which is available to authorized users.

## Background

Numerous chemical cosmetic products are used in hairdressing and beauty salons [1]. These products are used in facial cleansing, skin, nails and body hydrotherapy and care, anti-wrinkle treatments, pigmentation and acne treatment, make up, body and face massage, reflexology, aromatherapy, face and body hair removal, and hair styling and coloring services [2, 3]. These chemicals release volatile organic compounds (VOCs), including methacrylates, phthalates, formaldehyde, etc. and pollutants like ozone and carbon monoxide [4]. People who work in beauty salons and even their customers can be exposed to high concentrations of these compounds.

Skin and respiratory disorders, carcinogenicity, and reproductive and genotoxic effects have been associated with compounds released in beauty salons [5–8]. Salon personnel often complain about eye, nose, throat, lung, and skin irritation [9–11]. Thus, such high-risk environments need to be assessed for the types and concentrations of toxic air pollutants that result in human exposure.

Benzene, toluene, ethylbenzene, and xylene (BTEX) have adverse health effects such as cancer and probable neurological responses like weakness, loss of appetite, fatigue, confusion, and nausea [12]. The acute and chronic effects of formaldehyde include sensory irritation, reduced lung function, nasopharyngeal cancer, and myeloid leukemia [13]. Benzene, toluene, ethylbenzene, and xylene have been considered to be human carcinogen (group A), having inadequate information to assess carcinogenic potential, not classifiable as to human carcinogenicity (group D), and having inadequate data for carcinogenic potential, respectively [14–17]. Formaldehyde has been classified as a probable human carcinogen (group B1) by IRIS and as a human carcinogen (Group 1) by the International Agency for Research on Cancer (IARC) [18, 19]. Acute exposure to acetaldehyde causes irritation of the eyes, skin, and respiratory tract. Acetaldehyde is also considered as a probable human carcinogen (Group B2) by IRIS [20].

Several studies have been conducted to investigate indoor air quality in hairdressing and beauty salons. Goldin et al. (2014) measured total VOC (TVOC) concentrations, particulate matter with aerodynamic diameter less than 2.5 μm (PM_2.5_), and carbon dioxide (CO_2_) in nail salons in Boston, United States. They found that performing tasks increased the air pollutant concentrations, and ventilation improved indoor air quality [21]. In another study, concentrations of VOCs, formaldehyde, CO_2_, and phthalate esters were measured at hairdressing salons in Taipei [22]. They detected a wide range of concentrations in various salons. Tsigonia et al. (2010) measured VOCs and formaldehyde in beauty salons. The main VOCs found in the salons were aromatics (toluene, xylene), esters and ketones (ethyl acetate, acetone, etc.) used as solvents, and terpenes (pinene, limonene, camphor, menthol) to provide desired odors [4].

These studies have reported measurements, but there remain uncertainties as to the relationships between the toxic air pollutant concentrations and the different various cosmetic practices. Investigating these relationships will determine the high-risk treatments and at-risk workers and customers. The use of mitigation methods have also not been adequately examined. The effects of different types of ventilation and air purifiers needs to be studied. The results of these studies can support the design of strategies for reducing exposure in these occupational environments. This aims of this study were to measure the concentrations of benzene, toluene, ethylbenzene, xylene, formaldehyde, and acetaldehyde in 20 large beauty salons and to relate these concentrations to the different environmental and occupational characteristics of salons.

## Methods

### Study design

Tehran is the capital of Iran, and have about 9 million residents. This city is faced with serious problems in case of ambient air pollution [23–26]. There are reports of heavy use of cosmetics by Iranian women [27, 28]. Conventional cosmetics in Iran are imported mainly from China, Turkey, Korea, and England [29, 30].

Benzene, toluene, ethylbenzene, xylene (BTEX), formaldehyde and acetaldehyde were sampled from the indoor and outdoor air of 20 beauty salons during winter 2016–2017 in Tehran, Iran. The selected salons were located in different areas of the city. A questionnaire was completed by each salon owner to record the basic characteristics of salons, such as area (m^2^), number of active employees, type of ventilation, working hours, type of ongoing treatments, etc. Smoking was prohibited in each monitored beauty salon. Three samples each were collected from both the inside and outside air of each salon using active sampling methods during mornings. To assess human exposure, the samplers were placed in the height of 1.5 m of active salons, near the working area. In total, 360 samples were collected, 180 each for inside and outside spaces (3 × 60 for BTEX, formaldehyde and acetaldehyde). After sampling, the sampling cartridges were sealed with plastic or brass end caps, placed in a sealed plastic box at 4 °C, and then transported to the laboratory. All sampling and analysis were completed during a 3 month period.

Services with potential emission of VOC were categorized in three main groups; hair coloring (dyes, bleaches, etc.), nail treatment (lacquers, polishes, etc.), and hair styling (oils, ointments, brilliantines, creams, gels, products for waving and straightening, etc.). The number of customers receiving each of these three services was recorded during sampling interval. The ventilations system of each salon can be categorized into three groups; 1) fan and closed window, 2) fan and open window, and 3) air purifier. Two salons used air purifiers. The models used were AIRMEGA 300 (COWAY, Korea) and IQAir HealthPro Plus - New Edition (IQAir, Switzerland) that have an activated carbon filter and a gas phase filter to remove gaseous pollutants, respectively. The air purifiers were placed in the center of salons.

### Sampling and analysis

For BTEX, active sampling was performed using a pump (Universal 224-44MTX, SKC, USA) with a flow rate of 200 mL/min, and a solid sorbent tube (coconut shell charcoal, 100 mg/50 mg, 226–01 – SKC, USA) for 30 min. Three samples (each for 30 min) were collected sequentially indoors and outdoors. The sorbent from each tube was extracted using 1 mL CS_2_ (76.13 g/mol, Merck, Germany) and 30 min sonication. Gas chromatography/flame ionization detector (GC-FID: Agilent 7890B, Agilent Technologies, Waldbronn, Germany) was used to quantify the concentrations of BTEX. The sampling and analysis procedure implemented NIOSH method 1501 [31]. One μL samples were injected to the glass column with a 5:1 split ratio. Injection and detector temperature were 250 °C and 300 °C, respectively. The column temperature was held at 40 °C for 10 min, and then increased by 10 °C/min to 230 °C. The carrier gas was helium with a flow of 2.6 mL/min. The results of sequential samplings were averaged to obtain a single value for each salon.

Formaldehyde samples were taken using a cartridge containing XAD-2 coated with (2-hydroxymethyl) piperdine (226–118 – SKC, USA), and a pump with flow rate of 50 mL/min for 30 min. Formaldehyde was desorbed from the cartridge with 10 mL of carbonyl-free acetonitrile (41.05 g/mol, Merck, Germany) and 30 min sonication. Gas chromatography/flame ionization detector (GC-FID: Agilent 7890B, Agilent Technologies, Waldbronn, Germany) was used to measure the formaldehyde concentrations. The procedure fully implemented NIOSH method 2541 [31]. One μL of the samples were injected into the capillary column in splitless mode, and with split vent time of 30 s. Injection and detector temperature were 250 °C and 300 °C, respectively. Column temperature was held at 70 °C for 1 min, and then increased by 15 °C/min to 240 °C, and held for 10 min. Carrier gas was helium with flow of 1 mL/min, with makeup flow of 29 mL/min.

Acetaldehyde was sampled using a solid sorbent tube 2-(hydroxymethyl) piperidine (2-HMP) on XAD-2, (450 mg/225 mg, 226–27 – SKC, USA) and a pump with flow rate of 50 mL/min for 30 min. Desorption was done with 5 mL toluene (92.14 g/mol, Merck, Germany) and 60 min ultrasonic. Gas chromatography/flame ionization detector (GC-FID: Agilent 7890B, Agilent Technologies, Waldbronn, Germany) was used to measure the acetaldehyde concentrations. The procedure fully implemented NIOSH method 2538 [31]. One μL splitless injections were made into the fused-silica capillary. Injection and detector temperature were 250 °C and 300 °C, respectively. The column temperature was increased from 70 °C by 6 °C/min to 110 °C, and then by 30 °C to 260 °C. The carrier gas was helium with flow of 1 mL/min, with makeup flow of 29 mL/min.

### Quality control/quality assurance

The pump flowrate was calibrated before each sampling with a gas flow meter (Model 4140, TSI Inc., USA). Analytical instruments were calibrated using analytical grade reagents before each set of samples in reasonable concentration ranges for BTEX (1–50 μg/m^3^), formaldehyde (1–100 μg/m^3^), and acetaldehyde (1–100 μg/m^3^). For quality assurance, in 10% of samplings, two sets of equipment were placed at the place simultaneously, and duplicate samples taken to estimate instrument precision. Replicate samples were recorded in two beauty salons. This analysis showed good agreement between sampling devices and replicate samples (Pearson’s *r* > 0.97). Quality assurance procedures also included field, laboratory and solvent blanks to check for contamination. Blank samples showed negligible BTEX contamination.

### Risk assessment

The cancer risks from exposure to benzene, formaldehyde, and acetaldehyde and non-cancer risk of toluene, ethylbenzene, and xylene were estimated. The Additional file 1 provides the details of the health risk assessments. Body weight and inhalation rate values recommended by US EPA are 70 kg and 20 m^3^/day, respectively [32]. Considering 8-h working shifts per day and 30 vacation in each year, EF can be calculated (=52 × 6/3–30) to be 74 days. Also, ED was assumed to be 30 years. In addition, averaging time for 70 years were obtained 25,500 days.

Deterministic risk assessment considers worst case or conservative scenarios. Alternatively, stochastic risk assessments estimate the probability distributions of toxic compounds’ risk. Stochastic calculations treat some variables as random variables drawn from known probability distributions. ModelRisk (Vose Software) was used to simulate the distribution of risk based on the distribution of parameters used in the risk calculations by Monte Carlo analysis. The concentrations of the air pollutants were assumed to have log-normal distributions. Exposure frequency and exposure duration were considered to be distributed normally with the mean values of 74 days and 30 years, respectively. The minimum and maximum values were considered as 52 and 96 days for EF and 25 and 35 years for ED. CSFs for benzene and Reference concentrations (RfCs) for non-carcinogens were obtained from integrated risk information system (IRIS). According to this database, CSF for benzene, formaldehyde, and acetaldehyde are 0.029, 0.045, and 0.0077 1/(mg/kg.day), respectively. RfCs for chronic inhalation exposure of TEX and acetaldehyde compounds are 5.0, 1.0, and 0.1 mg/m^3^, respectively. These values were converted to mg/kg.day as the dose unit. Therefore, the doses of toluene, ethylbenzene, and xylene were calculated to be 1.43, 0.29, and 0.029 mg/kg.day, respectively. The number of random samples were set at 1000. The outcome of the analysis for each compound was a histogram, and the 95% CI were calculated for each probability distribution.

### Statistical analysis

All statistical analyses were performed using Sigma Plot 12. Kolmogorov-Smirnov and Levene tests were used to check the normality of data, and equality of variances, respectively. To compare indoor and outdoor concentrations of each pollutant, paired t-test was used for data with normal distributions and equal variances. The effect of ventilation type was investigated using one-way ANOVA and Holm-Sidak test. The correlations between the concentrations of the measured pollutants were assessed with Pearson’s correlations. Multiple regression analysis was used to assess the effect of number of customers receiving different services (hair coloring, nail treatment, and hair styling) on the indoor VOC concentrations. Insignificant variables were removed from the model backward stepwise, and only the significant independent variable(s) are reported. The relationships between surface area of salons, temperature, relative humidity, and pollutant concentrations were analyzed separately using simple linear regression. The detailed results of statistical analyses are presented in the Additional file 1.

## Results

Concentrations of BTEX, formaldehyde, and acetaldehyde were measured in 20 beauty salons, and several environmental and occupational factors were recorded simultaneously. The average air temperature and relative humidity were 21.8 °C and 29%, respectively. Table 1 presents some basic characteristics of BSs, number of cosmetologists, and number of customers receiving treatments with high VOCs potential during sampling.

**Table 1:** Characteristics of 20 beauty salons used in this study

Salon No.	Area (m^2^)	Working hour (h)	No. of cosmetologists	Hair coloring^a^	Nail treatment^a^	Hair styling^a^	Ventilation^b^
1	120	8	8	5	3	7	F
2	130	9	7	7	3	12	F
3	120	9	5	4	5	10	F
4	130	7	9	2	7	4	F + W
5	160	8	15	7	4	6	F
6	105	9	3	3	2	2	F + W
7	125	10	9	8	4	4	AP
8	110	8	9	5	8	2	F + W
9	95	9	7	7	5	7	F
10	100	7	8	8	6	5	F
11	95	9	8	7	9	6	F
12	95	11	8	9	6	4	F
13	100	8	10	8	8	9	F
14	110	10	12	5	4	8	F
15	105	8	10	5	5	6	F + W
16	105	8	11	8	4	7	F + W
17	130	9	14	7	3	6	AP
18	100	11	10	6	7	9	F
19	115	7	12	4	3	7	F + W
20	95	9	9	6	6	8	F

^a^Each comprises all the activities related to hair coloring, nail treatment, and hair styling

^b^*F* fan, *F + W* fan plus open window, *AP* air purifier

**Fig. 1:**
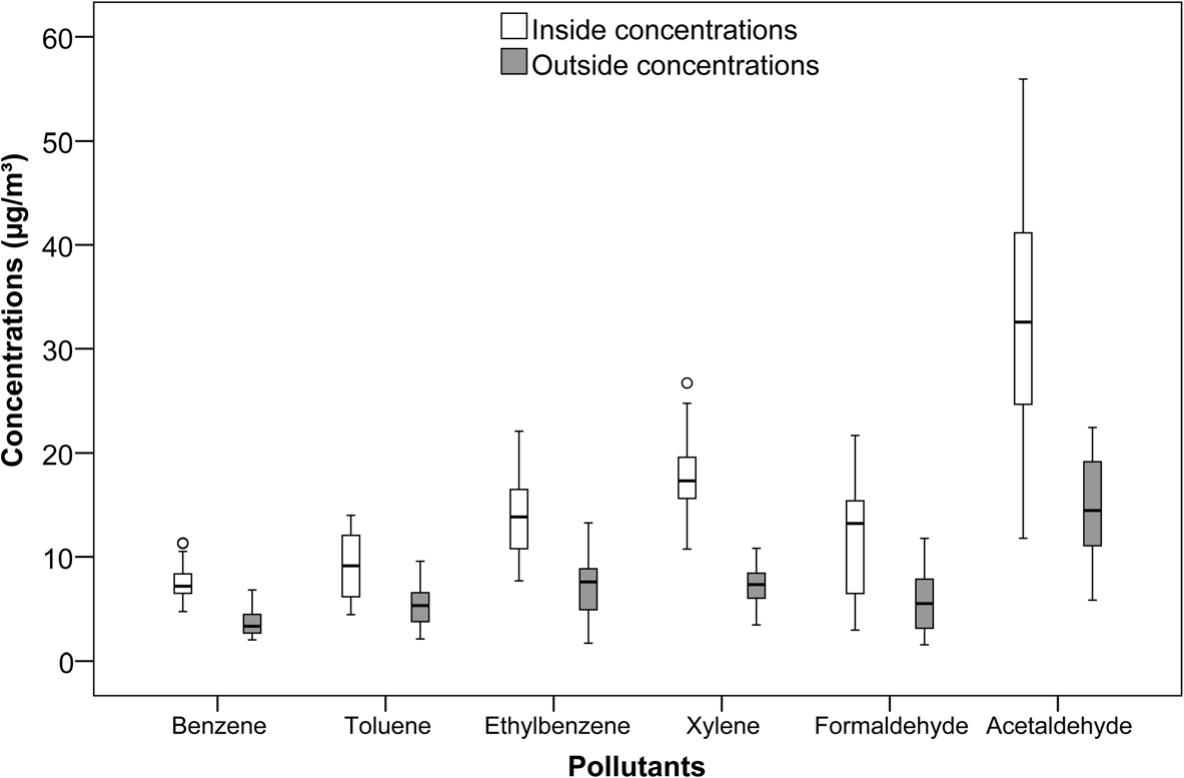
Descriptive statistics of inside and outside concentrations of air pollutants. Legend: The median, quartiles, minimum and maximum (whiskers), outliers (circles) and extreme values (asterisks) are shown in this Figure

Figure 1 presents box and whisker plots displaying the distributions of the pollutants inside and outside the beauty salons. Indoor concentrations of each pollutant were significantly higher than its outdoor concentrations (*p* < 0.05). The indoor to outdoor ratios for benzene, toluene, ethylbenzene, xylene, formaldehyde, and acetaldehyde were 2.04, 1.73, 2.01, 2.46, 2.11, and 2.21, respectively.

Concentrations of compounds in salons with different building characteristics were compared and the results are presented in Supplemental Material. The comparison between 3 types of ventilation mode showed that ventilations with air purifier, and fan and open window were more effective than just the fan (*p* < 0.05). No significant relationship was found between ventilation with air purifier, and fan and open window. In addition, floor area of salons did not affect the air pollutant concentrations (*p* > 0.05). Significant correlations were found between the concentrations of total VOCs (sum of all the measured compounds) and temperature (R^2^ = 0.71) and humidity (R^2^ = 0.74).

Table 2 presents the results of multiple regression about the relationship between the number of ongoing processes in beauty salons and concentrations of air pollutants. The results of multiple regression showed that concentrations of benzene and toluene were affected only by the number of hair dying treatments. Concentrations of xylene was affected only by the number of hair styling processes. And finally, the concentrations of formaldehyde were affected by either the number of hair stylings and nail treatments. The number of any processes had no effect the concentrations of acetaldehyde.

**Table 2:** Parameters affecting the indoor concentrations of air pollutants

	Parameter	Coefficient	Std. coefficient	Std. Error	*P*-value	R^2^
Benzene	Constant	3.786	–	1.163	–	0.387
	Dying	0.620	0.622	0.184	0.003	
Toluene	Constant	4.768	–	2.146	–	0.204
	Dying	0.729	0.452	0.339	0.046	
Ethylbenzene	–	–	–	–	–	–
Xylene	Constant	13.027	–	2.356	–	0.222
	Hair styling	0.772	0.471	0.341	0.036	
Formaldehyde	Constant	−2.232	–	4.265	–	0.415
	Nail	1.639	0.549	0.556	0.009	
	Hair styling	0.892	0.386	0.431	0.054	
Acetaldehyde	–	–	–	–	–	–

Table 3 presents the Pearson correlation coefficients between 6 investigated air pollutants. According to this table, only the correlation between formaldehyde and acetaldehyde (r = 0.65) was significant (*p*<0.05), indicating that 65% of formaldehyde and acetaldehyde variations are associated.

**Table 3:** Pearson’s correlation coefficients between air pollutants

Pollutants	Benzene	Ethylbenzene	Toluene	Xylene	Formaldehyde	Acetaldehyde
Benzene	1.00	−0.43	0.52	−0.04	−0.07	0.16
Ethylbenzene			−0.24	0.45	−0.04	−0.31
Toluene				0.27	0.02	0.13
Xylene					0.27	0.12
Formaldehyde						***0.65*** ^*a*^
Acetaldehyde						1.00

^a^*p*<0.05

**Fig. 2:**
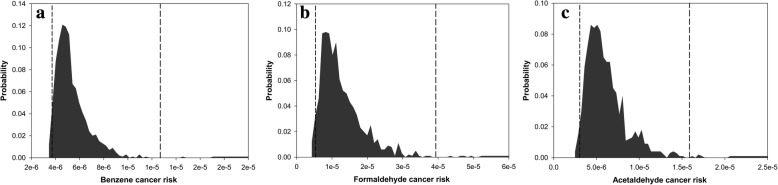
Probability distribution of cancer risk for (**a**) benzene, (**b**) formaldehyde, and (**c**) acetaldehyde Legend: The bars represent the probability of cancer risk, and the two vertical lines shows the confidence interval of 95%

**Fig. 3:**
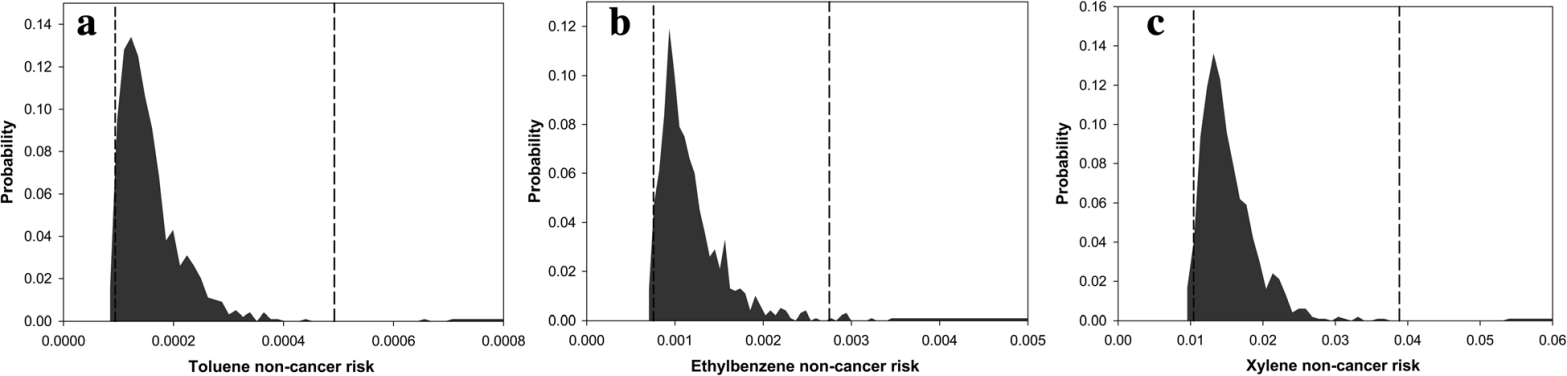
Probability distribution of hazard ratios for (**a**) toluene, (**b**) ethylbenzene, and (**c**) xylene Legend: The bars represent the probability of non-cancer risk, and the two vertical lines shows the confidence interval of 95%

According to deterministic risk assessment analyses, the cancer risks of benzene, formaldehyde and acetaldehyde were estimated to be 5.44 × 10^− 6^, 1.33 × 10^− 5^, and 6.26 × 10^− 6^. Hazard ratios for toluene, ethylbenzene, and xylene were 1.60 × 10^− 4^, 1.20 × 10^− 3^, and 1.54 × 10^− 2^, respectively. Figs. 2 and 3 show the results of the stochastic risk assessment for BTEX compounds, formaldehyde and acetaldehyde. The minimum cancer risks of benzene, formaldehyde and acetaldehyde were predicted to be 3.11 × 10^− 6^, 3.13 × 10^− 6^, and 2.12 × 10^− 6^, respectively. The maximum values of cancer risks for benzene, formaldehyde and acetaldehyde were 9.04 × 10^− 6^, 2.70 × 10^− 5^, and 1.12 × 10^− 5^, respectively. The minimum hazard ratios for toluene, ethylbenzene, and xylene were 7.12 × 10^− 6^, 6.45 × 10^− 4^, and 8.76 × 10^− 3^, respectively. The maximum values of hazard ratios predicted for toluene, ethylbenzene, and xylene were 2.57 × 10–4, 2.05 × 10^− 3^ and 2.49 × 10^− 2^, respectively.

## Discussion

The indoor concentrations of each pollutant were higher than the corresponding values in the local ambient air. This result was expected given that cosmetic products are known emission sources. The I/O ratios were between 1.7 and 2.4 for all measured pollutants. de Gennaro et al. (2014) found very high (> 10) indoor to outdoor (I/O) ratios of VOC concentrations in hair salons [33]. The lower ratios in the present study can be associated with the presence of ventilation systems. de Gennaro et al. (2014) did not discuss the ventilation in their measured salons. Goldin et al. (2014) measured total VOC concentrations in nail salons and reported the median concentration was 4800 ppb [21]. Tsigonia et al. (2010) examined VOCs in beauty salons, and reported that the major detected VOCs were aromatics (toluene, xylene), esters and ketones (ethyl acetate, acetone, etc.), terpenes (pinene, limonene, camphor, menthenol), and camphor. Formaldehyde concentrations were below detection limit of their method [4]. This difference with the present study may be due to differences in the cosmetic products in use and measurements were made in small salons with fewer customers. Chang et al. (2017) investigated indoor air of hairdressing salons in Taipei, and found 387 different ingredients. Their minimum and maximum formaldehyde concentrations were 12.40 and 1.04 × 10^3^ μg/m^3^, respectively [22]. In our study, all of the observed formaldehyde concentrations were lower than WHO guideline value of 100 μg/m^3^ for 30-min exposures. In case of benzene, no safe level of exposure has been recommended by WHO [12].

Beauty salons with better ventilations had lower concentrations of VOCs. Chang et al. (2017) reported high concentrations of CO_2_ in salons with poor ventilation [22]. Goldin et al. (2014) observed higher TVOC and PM_2.5_ concentrations in salons with less ventilation. It appears that salons with open doors, and table or roof fans had lower concentrations of pollutants compared to enclosed buildings with central ventilation systems [21].

Formaldehyde and acetaldehyde were correlated to each other, likely due to common sources. However, acetaldehyde was not related to any ongoing treatments. However, nail treatments and hair styling affected formaldehyde concentrations. An additional linear regression analysis was performed to investigate just the effect of hair styling, the variable that had the lowest *P*-value in the multiple regression analysis, on acetaldehyde concentration. The fit was marginally significant (*p* < 0.1). The intercept and slope for hair styling were 20.47 and 1.89, respectively. This shows that the number of hair styling treatments affects both formaldehyde and acetaldehyde concentrations, but in different statistical significance levels. In addition, Fig. 1 showed that acetaldehyde concentrations were higher than formaldehyde. This can be due to the content of cosmetic products. Additional studies should be conducted to explore the relationship between the ingredients of cosmetic products and toxic compounds in the air.

Temperature and relative humidity were positively correlated with the total VOC concentrations in accordance with prior literature [34, 35]. Higher temperatures increase the evaporation of VOCs from cosmetic products [36]. Therefore, in order to decrease the concentrations of VOCs in beauty salons, the optimum conditions in case of temperature and humidity can be provided. However, Quach et al. (2011) report that temperature was weakly correlated with toluene and isopropyl acetate concentrations. Relative humidity had no relationship with measured concentrations for any of the compounds [37].

Significant relationships were found between compound concentrations and the number of ongoing treatments. The results found relationships between benzene-hair dying, toluene-hair dying, xylene-hair styling, formaldehyde-nail treatment, and formaldehyde-hair styling. Goldin et al. (2014) reported higher TVOC concentrations were observed during nail treatments. However, TVOCs concentrations were independent of the number of ongoing nail treatments [21]. Quach et al. (2011) reported that workers who performed pedicures were more likely to be exposed to higher ethyl acetate values compared with those who applied silk nails and acrylic nails. They found that the number of permanent wave treatments and the number of workers were associated with formaldehyde concentrations [37].

According to previous studies, compounds with an attributable cancer risk more than 1 × 10^− 4^ were defined as a “definite risk”, those between 1 × 10^− 5^ and 1 × 10^− 4^ were “probable risk”, and between 1 × 10^− 5^ and 1 × 10^− 6^ was a “possible risk”. A cancer risk less than 1 × 10^− 6^ is recommended by USEPA as an “acceptable risk” [38]. In this study, minimum, average, and maximum carcinogen risks for benzene, formaldehyde, and acetaldehyde were exceeded 1 × 10^− 6^. Hence, these compounds represent a possible cancer risk in the beauty salons but does not pose a significant risk. To assess the non-carcinogenic effects of the TEX compounds, an HR below 1 should be considered to be as a negligible risk [39]. The findings in this study showed average, minimum, and maximum non-carcinogenic risks of toluene, ethylbenzene, and xylene in beauty salons were less than one. Therefore, they can be considered to have negligible non-carcinogenic risks. In addition to the risk attributed to each compound, the total cumulative non-cancer risk can be an important value. By aggregating all the individual values, total cumulative non-cancer risk was 1.70 × 10^− 2^, that is still less than one.

## Conclusions

High inside to outside ratio of air pollutant concentrations demonstrated that the indoor activities were VOC sources. Significant relationships between the concentrations of some compounds (i.e. benzene, toluene, ethylbenzene, and formaldehyde) and the number of different treatments identified possible sources for these compounds. Relationships between air pollutant concentrations and the salon characteristics were analyzed, and effective ventilation was found to reduce exposure. Chronic exposure to a mixture of air pollutants can impose greater adverse health effects rather than single exposures. Thus, to protect the workers, controlling ventilation, it is possible to reduce indoor pollutant concentrations. It would also be possible to use products with little or none of these toxic species as ingredients. To improve health conditions in beauty salons, air quality guidelines or a mandatory occupational regulatory framework is needed. In addition, identifying and prohibiting cosmetic products with potentially toxic emissions could reduce VOC exposures to both workers and customers.

## Additional file

Additional file 1: More details of the chemical analyses and the detailed results of the statistical analyses are presented in the Additional file; other information is also available from the corresponding author upon reasonable request. (DOCX 70 kb)

## Abbreviations

BTEX: Benzene, toluene, ethylbenzene, xylene
CSF: Cancer slope factor
ED: Exposure duration
EF: Exposure frequency
GC-FID: Gas chromatography-flame ionization detector
RfC: Reference concentration
RfD: Reference dose
TVOC: Total volatile organic compound
VOC: Volatile organic compound

## References

[1] Leino T (1999). Working conditions and health in hairdressing salons. Appl Occup Environ Hyg.

[2] Labrèche F, Forest J, Trottier M, Lalonde M, Simard R (2003). Characterization of chemical exposures in hairdressing salons. Appl Occup Environ Hyg.

[3] Ronda E, Hollund BE, Moen BE (2009). Airborne exposure to chemical substances in hairdresser salons. Environ Monit Assess.

[4] Tsigonia A, Lagoudi A, Chandrinou S, Linos A, Evlogias N, Alexopoulos EC (2010). Indoor air in beauty salons and occupational health exposure of cosmetologists to chemical substances. Int J Environ Res Public Health.

[5] Leino T, Tammilehto L, Hytonen M (1999). Occupational skin and respiratory diseases among hairdressers. Occupat Health Industr Med.

[6] Halliday-Bell JA, Gissler M, Jaakkola JJ (2009). Work as a hairdresser and cosmetologist and adverse pregnancy outcomes. Occup Med.

[7] Galiotte MP, Kohler P, Mussi G, Figaro Gattás GJ (2008). Assessment of occupational genotoxic risk among Brazilian hairdressers. Ann Occup Hyg.

[8] Czene K, Tiikkaja S, Hemminki K (2003). Cancer risks in hairdressers: assessment of carcinogenicity of hair dyes and gels. Int J Cancer.

[9] Leino T, Tammilehto L, Luukkonen R, Nordman H (1998). Self reported respiratory symptoms and diseases among hairdressers. Occupat Health Industr Med.

[10] Palmer A, Renzetti AD, Gillam D (1979). Respiratory disease prevalence in cosmetologists and its relationship to aerosol sprays. Environ Res.

[11] Uter W, Gefeller O, Schwanitz H (1995). The influence of skin sensitivity (atopy) and skin protection on the development of hand eczema in hairdressers-first results of a prospective cohort study. Allergologie.

[12] World Health Organization. WHO Guidelines for indoor air quality: selected pollutants. København: WHO Regional Office for Europe; 2010. 23741784

[13] Mandin C, Trantallidi M, Cattaneo A, Canha N, Mihucz VG, Szigeti T, Mabilia R, Perreca E, Spinazzè A, Fossati S (2017). Assessment of indoor air quality in office buildings across Europe–the OFFICAIR study. Sci Total Environ.

[14] IRIS (2003). Chemical assessment summary: benzene; CASRN 71–43-2**.** U.S..

[15] IRIS (2005). Chemical Assessment Summary: toluene; CASRN 108–88-3**.** U.S..

[16] IRIS (1988). Chemical Assessment Summary: Ethylbenzene; CASRN 100–41-4**.** U.S..

[17] IRIS (2003). Chemical Assessment Summary: xylenes; CASRN 1330-20-7**.** U.S..

[18] U.S.EPA (1989). Integrated risk information system (IRIS) on formaldehyde.

[19] IARC (2006). Formaldehyde, 2-butoxyethanol and 1-tert-butoxypropan-2-ol. IARC Monogr Eval Carcinog Risks Hum.

[20] U.S.EPA (1999). Integrated risk information system (IRIS) on acetaldehyde.

[21] Goldin LJ, Ansher L, Berlin A, Cheng J, Kanopkin D, Khazan A, Kisivuli M, Lortie M, Peterson EB, Pohl L (2014). Indoor air quality survey of nail salons in Boston. J Immigr Minor Health.

[22] Chang CJ, Cheng SF, Chang PT, Tsai SW (2018). Indoor air quality in hairdressing salons in Taipei. Indoor Air.

[23] Hadei M, Hopke PK, Hashemi Nazari SS, Yarahmadi M, Shahsavani A, Alipour MR (2017). Estimation of mortality and hospital admissions attributed to criteria air pollutants in Tehran Metropolis, Iran (2013-2016). Aerosol Air Qual Res..

[24] Shahsavani A, Yarahmadi M, Hadei M, Sowlat MH, Naddafi K (2017). Elemental and carbonaceous characterization of TSP and PM10 during middle eastern dust (MED) storms in Ahvaz, Southwestern Iran. Environ Monit Assess.

[25] Bakhtiari R, Hadei M, Hopke PK, Shahsavani A, Rastkari N, Kermani M, Yarahmadi M, Ghaderpoori A (2018). Investigation of in-cabin volatile organic compounds (VOCs) in taxis; influence of vehicle's age, model, fuel, and refueling. Environ Pollut.

[26] Khamutian R, Najafi F, Soltanian M, Shokoohizadeh MJ, Poorhaghighat S, Dargahi A, Sharafi K, Afshari A (2015). The association between air pollution and weather conditions with increase in the number of admissions of asthmatic patients in emergency wards: a case study in Kermanshah. Med J Islam Repub Iran.

[27] Dehghani R, Talaee R, Sehat M, Ghamsari NN, Mesgari L (2017). Investigating the influence of mass media on cosmetics usage among women in Kashan during 2015. Iran J Health Safety Environ.

[28] Dehghani R, Talaee R, Sehat M, Ghamsari NN, Mesgari L (2017). Surveying the rate of using cosmetics among the Kashan's women. J Biol Today's World.

[29] Karimi G, Ziarati P (2015). Heavy metal contamination of popular nail polishes in Iran. Iranian Journal of Toxicology.

[30] Mousavi Z, Ziarati P, Shariatdoost A (2013). Determination and safety assessment of lead and cadmium in eye shadows purchased in local market in Tehran. J Environ Anal Toxicol.

[31] Eller PM, Cassinelli ME. NIOSH manual of analytical methods: Diane Publishing; 1994.

[32] EPA A (1989). Risk assessment guidance for superfund. Volume I: human health evaluation manual (part a). EPA/540/1–89/002.

[33] de Gennaro G, de Gennaro L, Mazzone A, Porcelli F, Tutino M (2014). Indoor air quality in hair salons: screening of volatile organic compounds and indicators based on health risk assessment. Atmos Environ.

[34] Wiglusz R, Sitko E, Nikel G, Jarnuszkiewicz I, Igielska B (2002). The effect of temperature on the emission of formaldehyde and volatile organic compounds (VOCs) from laminate flooring—case study. Build Environ.

[35] Markowicz P, Larsson L (2015). Influence of relative humidity on VOC concentrations in indoor air. Environ Sci Pollut Res.

[36] Wang R, Moody RP, Koniecki D, Zhu J (2009). Low molecular weight cyclic volatile methylsiloxanes in cosmetic products sold in Canada: implication for dermal exposure. Environ Int.

[37] Quach Thu, Gunier Robert, Tran Alisha, Von Behren Julie, Doan-Billings Phuong-An, Nguyen Kim-Dung, Okahara Linda, Lui Benjamin Yee-Bun, Nguyen Mychi, Huynh Jessica, Reynolds Peggy (2011). Characterizing Workplace Exposures in Vietnamese Women Working in California Nail Salons. American Journal of Public Health.

[38] Robson MG, Toscano WA. Risk Assessment for Environmental Health: John Wiley & Sons; 2007.

[39] Ramírez N, Cuadras A, Rovira E, Borrull F, Marcé RM (2012). Chronic risk assessment of exposure to volatile organic compounds in the atmosphere near the largest Mediterranean industrial site. Environ Int.

