# Supplementary material for: Indoor concentrations of VOCs in beauty salons; association with cosmetic practices and health risk assessment
Source: J Occup Med Toxicol. 2018 Sep 27;13:30. doi: 10.1186/s12995-018-0213-x (PMC6161385; doi:10.1186/s12995-018-0213-x)
Supplement: Supplementary file 1 — More details of the chemical analyses and the detailed results of the statistical analyses are presented in the Additional file; other information is also available from the corresponding author upon reasonable request. (DOCX 70 kb) [file 12995_2018_213_MOESM1_ESM.docx]

Archives of Environmental Contamination and Toxicology

**Supplementary material for:**

**Indoor Concentrations of VOCs in Beauty Salons, the Association with Different Cosmetic Practices and Health Risk Assessment**

Mostafa Hadei ^1^, Philip K. Hopke ^2^, Abbas Shahsavani ^3,4^, Mahbobeh Moradi ^4^

Maryam Yarahmadi ^5^, , Baharan Emam ^4^, Noushin Rastkari^4^

^1^ Research Center for Environmental Determinants of Health (RCEDH), Kermanshah University of Medical Sciences, Kermanshah, Iran.

Email: [Mostafa.hadei@gmail.com](mailto:Mostafa.hadei@gmail.com)

^2^ Department of Public Health Sciences, University of Rochester School of Medicine and Dentistry, Rochester, NY 14642 USA

^3^ Environmental and Occupational Hazards Control Research Center, Shahid Beheshti University of Medical Science, Tehran, Iran

^4^ Department of Environmental Health Engineering, School of Public Health, Shahid Beheshti University of Medical Science, Tehran, Iran

^5^ Environmental and Occupational Health Center, Ministry of Health and Medical Education, Tehran, Iran

*Corresponding Author: Abbas Shahsavani

Email: [ashahsavani@gmail.com](mailto:ashahsavani@gmail.com)

Tel: +98 9102006560; Fax: +98 21 22432037

Address: Shahid Beheshti University of Medical Science, Daneshjo Street, Velenjak Square, Tehran, Iran

**Procedure for Health Risk Assessment**

Cancer risk for carcinogen compounds can be calculated by the following equation:

Cancer risk = I × CSF Equation (1)

Which I and CSF are the daily intake (mg/kg. day) and cancer slope factor (1/(mg/kg.day)) for the interest compound, respectively.

Non-carcinogenic risk can be determined using hazard ratio (HR):

HR = I/ RfD Equation (2)

Where I and RfD are the daily intake (mg/kg. day) and reference dose (RfD) as the daily intake of a compound that should not contributes to an adverse health effect (LaGrega et al., 2010). The daily intake is calculated from the following equation:

$I=\frac{C\times CF\times IR\times EF\times ED}{BW\times AT}$ Equation (3)

Where C, CF, IR, EF, ED, BW and AT are the concentration of compound (μg/m^3^), the conversion factor (mg/μg), inhalation rate (m^3^/day), exposure frequency (day/year), exposure duration (year), body weight (kg) and averaging time (day), respectively.

**The results of statistical analysis**

**Descriptive statistics**

**Column Size Missing Mean Std Dev Std. Error C.I. of Mean**

Ben-In 20 0 7.536 1.871 0.418 0.876

Ben-Out 20 0 3.669 1.461 0.327 0.684

EB-In 20 0 14.001 4.211 0.942 1.971

EB-Out 20 0 6.966 3.040 0.680 1.423

To-In 20 0 9.179 3.030 0.678 1.418

To-Out 20 0 5.313 1.980 0.443 0.927

OX-In 20 0 6.783 1.915 0.428 0.896

OX-Out 20 0 3.009 0.904 0.202 0.423

MX-In 20 0 11.225 2.570 0.575 1.203

MX-Out 20 0 4.298 1.061 0.237 0.497

Form-In 20 0 11.882 5.885 1.316 2.754

Form-Out 20 0 5.642 2.965 0.663 1.388

Acet-In 20 0 32.671 12.592 2.816 5.893

Acet-Out 20 0 14.761 5.281 1.181 2.472

Area 20 0 112.250 16.739 3.743 7.834

Workers 20 0 9.200 2.802 0.627 1.311

Dying 20 0 6.050 1.877 0.420 0.879

Nail 20 0 4.500 1.357 0.303 0.635

Shenyon 20 0 6.450 2.544 0.569 1.191

**Column Range Max Min Median 25% 75%**

Ben-In 6.599 11.329 4.730 7.169 6.381 8.503

Ben-Out 4.800 6.798 1.998 3.311 2.644 4.744

EB-In 14.421 22.102 7.681 13.840 10.400 16.934

EB-Out 11.587 13.261 1.674 7.580 4.658 8.855

To-In 9.573 13.998 4.425 9.132 6.139 12.117

To-Out 7.487 9.570 2.082 5.302 3.564 6.613

OX-In 6.315 9.435 3.120 6.884 5.596 8.674

OX-Out 3.353 4.456 1.103 3.138 2.506 3.680

MX-In 9.634 17.255 7.621 10.531 9.486 11.912

MX-Out 4.025 6.358 2.333 4.256 3.587 5.142

Form-In 18.757 21.692 2.936 13.211 6.414 15.854

Form-Out 10.246 11.771 1.525 5.498 3.106 8.024

Acet-In 44.137 55.925 11.788 32.564 24.354 41.411

Acet-Out 16.634 22.456 5.822 14.456 10.708 19.233

Area 65.000 160.000 95.000 107.500 100.000 123.750

Workers 12.000 15.000 3.000 9.000 8.000 10.750

Dying 7.000 9.000 2.000 6.500 5.000 7.750

Nail 5.000 7.000 2.000 4.500 3.250 5.000

Shenyon 10.000 12.000 2.000 6.500 4.250 8.000

**Column Skewness Kurtosis K-S Dist. K-S Prob. SWilk W SWilk Prob**

Ben-In 0.825 0.0817 0.209 0.022 0.914 0.076

Ben-Out 0.924 -0.195 0.200 0.036 0.887 0.024

EB-In 0.448 -0.655 0.146 0.310 0.949 0.355

EB-Out 0.178 -0.221 0.120 0.581 0.967 0.698

To-In -0.0624 -1.285 0.139 0.376 0.942 0.261

To-Out 0.284 -0.191 0.120 0.576 0.973 0.813

OX-In -0.425 -0.846 0.123 0.542 0.942 0.261

OX-Out -0.541 -0.296 0.117 0.611 0.960 0.549

MX-In 1.006 0.645 0.231 0.007 0.893 0.031

MX-Out 0.371 -0.335 0.132 0.446 0.966 0.671

Form-In 0.0987 -1.062 0.148 0.289 0.939 0.229

Form-Out 0.365 -0.797 0.145 0.318 0.955 0.444

Acet-In 0.0688 -0.636 0.0700 0.857 0.978 0.907

Acet-Out -0.134 -1.026 0.112 0.659 0.952 0.399

Area 1.265 1.945 0.168 0.146 0.873 0.013

Workers 0.0434 0.726 0.138 0.388 0.967 0.701

Dying -0.504 -0.436 0.194 0.048 0.945 0.295

Nail 0.211 -0.383 0.156 0.220 0.943 0.276

Shenyon 0.118 0.0854 0.130 0.470 0.972 0.796

**Column Sum Sum of Squares**

Ben-In 150.720 1202.343

Ben-Out 73.383 309.831

EB-In 280.016 4257.363

EB-Out 139.325 1146.213

To-In 183.574 1859.402

To-Out 106.266 639.139

OX-In 135.666 989.964

OX-Out 60.187 196.664

MX-In 224.500 2645.534

MX-Out 85.967 390.918

Form-In 237.631 3481.347

Form-Out 112.832 803.587

Acet-In 653.426 24360.668

Acet-Out 295.229 4887.910

Area 2245.000 257325.000

Workers 184.000 1842.000

Dying 121.000 799.000

Nail 90.000 440.000

Shenyon 129.000 955.000

**Formaldehyde regression**

**Backward Stepwise Regression:**

Dependent Variable:Form-In

F-to-Enter: 4.000 P = 0.060

F-to-Remove: 3.900 P = 0.063

Step 0:

Standard Error of Estimate = 4.895

Analysis of Variance:

**Group DF SS MS F P**

Regression 3 274.589 91.530 3.820 0.031

Residual 16 383.343 23.959

Variables in Model

**Group Coef. Std. Coeff. Std. Error F-to-Remove P**

Constant -2.936 5.169

Dying 0.158 0.0504 0.614 0.0663 0.800

Nail 1.613 0.540 0.580 7.733 0.013

Shenyon 0.873 0.378 0.449 3.781 0.070

Variables not in Model

**Group F-to-Enter P**

Step 1: Dying Removed

R = 0.644 Rsqr = 0.415 Adj Rsqr = 0.346

Standard Error of Estimate = 4.758

Analysis of Variance:

**Group DF SS MS F P**

Regression 2 273.001 136.501 6.028 0.011

Residual 17 384.930 22.643

Variables in Model

**Group Coef. Std. Coeff. Std. Error F-to-Remove P**

Constant -2.232 4.265

Nail 1.639 0.549 0.556 8.693 0.009

Shenyon 0.892 0.386 0.431 4.294 0.054

Summary Table

**Step # Vars. Entered Vars. Removed R RSqr Delta RSqr Vars in Model**

1 Dying 0.644 0.415 0.415 2

The dependent variable Form-In can be predicted from a linear combination of the independent variables:

**P**

Nail 0.009

Shenyon 0.054

Normality Test (Shapiro-Wilk) Passed (P = 0.660)

Constant Variance Test: Passed (P = 0.255)

Power of performed test with alpha = 0.050: 0.884

**Benzene regression**

**Backward Stepwise Regression:** Thursday, August 31, 2017, 8:01:30 PM

**Data source:** Data 1 in Salons.JNB

Dependent Variable:Ben-In

F-to-Enter: 4.000 P = 0.060

F-to-Remove: 3.900 P = 0.063

Step 0:

Standard Error of Estimate = 1.530

Analysis of Variance:

**Group DF SS MS F P**

Regression 3 29.075 9.692 4.142 0.024

Residual 16 37.435 2.340

Variables in Model

**Group Coef. Std. Coeff. Std. Error F-to-Remove P**

Constant 4.156 1.615

Dying 0.635 0.637 0.192 10.948 0.004

Nail -0.184 -0.193 0.181 1.025 0.326

Shenyon 0.0737 0.100 0.140 0.276 0.607

Variables not in Model

**Group F-to-Enter P**

Step 1: Shenyon Removed

R = 0.654 Rsqr = 0.427 Adj Rsqr = 0.360

Standard Error of Estimate = 1.497

Analysis of Variance:

**Group DF SS MS F P**

Regression 2 28.430 14.215 6.346 0.009

Residual 17 38.081 2.240

Variables in Model

**Group Coef. Std. Coeff. Std. Error F-to-Remove P**

Constant 4.584 1.365

Dying 0.651 0.654 0.185 12.377 0.003

Nail -0.194 -0.204 0.176 1.209 0.287

Variables not in Model

**Group F-to-Enter P**

Shenyon 0.276 0.606

Step 2: Nail Removed

R = 0.622 Rsqr = 0.387 Adj Rsqr = 0.353

Standard Error of Estimate = 1.505

Analysis of Variance:

**Group DF SS MS F P**

Regression 1 25.723 25.723 11.352 0.003

Residual 18 40.788 2.266

Variables in Model

**Group Coef. Std. Coeff. Std. Error F-to-Remove P**

Constant 3.786 1.163

Dying 0.620 0.622 0.184 11.352 0.003

Variables not in Model

**Group F-to-Enter P**

Nail 1.209 0.286

Shenyon 0.408 0.531

Summary Table

**Step # Vars. Entered Vars. Removed R RSqr Delta RSqr Vars in Model**

1 Shenyon 0.654 0.427 0.427 2

2 Nail 0.622 0.387 -0.0407 1

The dependent variable Ben-In can be predicted from a linear combination of the independent variables:

**P**

Dying 0.003

The following variables did not significantly add to the ability of the equation to predict Ben-In and were not included in the final equation: Nail Shenyon

Normality Test (Shapiro-Wilk) Passed (P = 0.370)

Constant Variance Test: Passed (P = 0.198)

Power of performed test with alpha = 0.050: 0.851

**Toluene regression**

**Backward Stepwise Regression:** Thursday, August 31, 2017, 8:02:18 PM

**Data source:** Data 1 in Salons.JNB

Dependent Variable:To-In

F-to-Enter: 4.000 P = 0.060

F-to-Remove: 3.900 P = 0.063

Step 0:

Standard Error of Estimate = 2.586

Analysis of Variance:

**Group DF SS MS F P**

Regression 3 67.413 22.471 3.359 0.045

Residual 16 107.028 6.689

Variables in Model

**Group Coef. Std. Coeff. Std. Error F-to-Remove P**

Constant 5.394 2.731

Dying 0.755 0.468 0.324 5.416 0.033

Nail -0.515 -0.335 0.307 2.817 0.113

Shenyon 0.286 0.240 0.237 1.448 0.246

Variables not in Model

**Group F-to-Enter P**

Step 1: Shenyon Removed

R = 0.575 Rsqr = 0.331 Adj Rsqr = 0.252

Standard Error of Estimate = 2.620

Analysis of Variance:

**Group DF SS MS F P**

Regression 2 57.730 28.865 4.204 0.033

Residual 17 116.711 6.865

Variables in Model

**Group Coef. Std. Coeff. Std. Error F-to-Remove P**

Constant 7.051 2.390

Dying 0.819 0.508 0.324 6.388 0.022

Nail -0.555 -0.361 0.309 3.227 0.090

Variables not in Model

**Group F-to-Enter P**

Shenyon 1.448 0.245

Step 2: Nail Removed

R = 0.452 Rsqr = 0.204 Adj Rsqr = 0.160

Standard Error of Estimate = 2.778

Analysis of Variance:

**Group DF SS MS F P**

Regression 1 35.576 35.576 4.611 0.046

Residual 18 138.864 7.715

Variables in Model

**Group Coef. Std. Coeff. Std. Error F-to-Remove P**

Constant 4.768 2.146

Dying 0.729 0.452 0.339 4.611 0.046

Variables not in Model

**Group F-to-Enter P**

Nail 3.227 0.089

Shenyon 1.755 0.202

Summary Table

**Step # Vars. Entered Vars. Removed R RSqr Delta RSqr Vars in Model**

1 Shenyon 0.575 0.331 0.331 2

2 Nail 0.452 0.204 -0.127 1

The dependent variable To-In can be predicted from a linear combination of the independent variables:

**P**

Dying 0.046

The following variables did not significantly add to the ability of the equation to predict To-In and were not included in the final equation: Nail Shenyon

Normality Test (Shapiro-Wilk) Passed (P = 0.924)

Constant Variance Test: Passed (P = 0.772)

Power of performed test with alpha = 0.050: 0.519

**Xylene regression**

**Backward Stepwise Regression:** Thursday, August 31, 2017, 8:03:12 PM

**Data source:** Data 1 in Salons.JNB

Dependent Variable:Xyl-In

F-to-Enter: 4.000 P = 0.060

F-to-Remove: 3.900 P = 0.063

Step 0:

Standard Error of Estimate = 3.758

Analysis of Variance:

**Group DF SS MS F P**

Regression 3 104.617 34.872 2.469 0.099

Residual 16 225.940 14.121

Variables in Model

**Group Coef. Std. Coeff. Std. Error F-to-Remove P**

Constant 9.295 3.969

Dying 0.0297 0.0134 0.471 0.00398 0.950

Nail 0.648 0.306 0.445 2.116 0.165

Shenyon 0.811 0.494 0.345 5.528 0.032

Variables not in Model

**Group F-to-Enter P**

Step 1: Dying Removed

R = 0.562 Rsqr = 0.316 Adj Rsqr = 0.236

Standard Error of Estimate = 3.646

Analysis of Variance:

**Group DF SS MS F P**

Regression 2 104.561 52.280 3.933 0.039

Residual 17 225.996 13.294

Variables in Model

**Group Coef. Std. Coeff. Std. Error F-to-Remove P**

Constant 9.428 3.268

Nail 0.653 0.308 0.426 2.349 0.144

Shenyon 0.814 0.497 0.330 6.090 0.025

Variables not in Model

**Group F-to-Enter P**

Dying 0.00398 0.950

Step 2: Nail Removed

R = 0.471 Rsqr = 0.222 Adj Rsqr = 0.179

Standard Error of Estimate = 3.780

Analysis of Variance:

**Group DF SS MS F P**

Regression 1 73.337 73.337 5.132 0.036

Residual 18 257.220 14.290

Variables in Model

**Group Coef. Std. Coeff. Std. Error F-to-Remove P**

Constant 13.027 2.356

Shenyon 0.772 0.471 0.341 5.132 0.036

Variables not in Model

**Group F-to-Enter P**

Dying 0.0930 0.764

Nail 2.349 0.143

Summary Table

**Step # Vars. Entered Vars. Removed R RSqr Delta RSqr Vars in Model**

1 Dying 0.562 0.316 0.316 2

2 Nail 0.471 0.222 -0.0945 1

The dependent variable Xyl-In can be predicted from a linear combination of the independent variables:

**P**

Shenyon 0.036

The following variables did not significantly add to the ability of the equation to predict Xyl-In and were not included in the final equation: Dying Nail

Normality Test (Shapiro-Wilk) Passed (P = 0.485)

Constant Variance Test: Failed (P = 0.042)

Power of performed test with alpha = 0.050: 0.559

**Spearman's correlation**

**Spearman Rank Order Correlation** Thursday, July 26, 2018, 7:12:28 AM

**Data source:** Data 1 in Salons 2 (Hopke Suggestions).JNB

Cell Contents:

Correlation Coefficient

P Value

Number of Samples

**EB-In To-In Xyl-In Form-In Acet-In**

**Ben-In -0.366 0.423 0.0459 0.0211 0.0693**

**0.109 0.0623 0.841 0.927 0.767**

**20 20 20 20 20**

**EB-In -0.268 0.465 -0.0511 -0.246**

**0.249 0.0385 0.826 0.289**

**20 20 20 20**

**To-In 0.101 0.129 0.108**

**0.667 0.581 0.644**

**20 20 20**

**Xyl-In 0.162 0.0564**

**0.488 0.807**

**20 20**

**Form-In 0.648**

**0.00196**

**20**

**Acet-In**

**The pair(s) of variables with positive correlation coefficients and P values below 0.050 tend to increase together. For the pairs with negative correlation coefficients and P values below 0.050, one variable tends to decrease while the other increases. For pairs with P values greater than 0.050, there is no significant relationship between the two variables.**

**Effect of ventilation**

**One Way Analysis of Variance** Monday, September 11, 2017, 8:06:14 PM

**Data source:** Data 1 in Salons.JNB

Dependent Variable: Total

**Normality Test (Kolmogorov-Smirnov)** Passed (P = 0.081)

**Equal Variance Test:** Passed (P = 0.146)

**Group Name N Missing Mean Std Dev SEM**

0.000 12 0 104.267 14.897 4.300

1.000 6 0 81.487 6.972 2.846

2.000 2 0 62.707 3.580 2.532

**Source of Variation DF SS MS F P**

Between Groups 2 4152.396 2076.198 13.087 <0.001

Residual 17 2697.073 158.651

Total 19 6849.469

The differences in the mean values among the treatment groups are greater than would be expected by chance; there is a statistically significant difference (P = <0.001).

Power of performed test with alpha = 0.050: 0.990

All Pairwise Multiple Comparison Procedures (Holm-Sidak method):

Overall significance level = 0.05

Comparisons for factor: **EC-Fan**

**Comparison Diff of Means t P P<0.050**

0.000 vs. 2.000 41.560 4.320 0.001 Yes

0.000 vs. 1.000 22.780 3.617 0.004 Yes

1.000 vs. 2.000 18.780 1.826 0.085 No

**Effect of Area**

**Linear Regression** Sunday, September 10, 2017, 12:39:31 AM

**Data source:** Data 1 in Salons.JNB

Total = 148.651 - (0.493 * Area )

N = 20

R = 0.435 Rsqr = 0.189 Adj Rsqr = 0.144

Standard Error of Estimate = 17.566

**Coefficient Std. Error t P**

Constant 148.651 27.307 5.444 <0.001

Area -0.493 0.241 -2.049 0.055

Analysis of Variance:

**DF SS MS F P**

Regression 1 1295.575 1295.575 4.199 0.055

Residual 18 5553.895 308.550

Total 19 6849.469 360.498

Normality Test (Shapiro-Wilk) Passed (P = 0.324)

Constant Variance Test: Passed (P = 0.117)

Power of performed test with alpha = 0.050: 0.485

The power of the performed test (0.485) is below the desired power of 0.800.

Less than desired power indicates you are less likely to detect a difference when one actually exists. Negative results should be interpreted cautiously.

**Correlation of Temperature and humiditiy**

**Pearson Product Moment Correlation** Wednesday, September 13, 2017, 1:45:03 AM

**Data source:** Data 1 in Salons.JNB

Cell Contents:

Correlation Coefficient

P Value

Number of Samples

**Temp. Humidity (%)**

Total 0.845 0.858

0.00000279 0.00000135

20 20

Temp. 0.715

0.000393

20

Humidity (%)

The pair(s) of variables with positive correlation coefficients and P values below 0.050 tend to increase together. For the pairs with negative correlation coefficients and P values below 0.050, one variable tends to decrease while the other increases. For pairs with P values greater than 0.050, there is no significant relationship between the two variables.

**Pearson's correlation**

**Pearson Product Moment Correlation** Wednesday, September 13, 2017, 1:46:12 AM

**Data source:** Data 1 in Salons.JNB

Cell Contents:

Correlation Coefficient

P Value

Number of Samples

**EB-In To-In Form-In Acet-In Xyl-In Temp. Humidity (%)**

Ben-In -0.432 0.522 -0.0690 0.159 -0.0430 0.304 0.211

0.0570 0.0182 0.773 0.502 0.857 0.192 0.372

20 20 20 20 20 20 20

EB-In -0.238 -0.0408 -0.310 0.448 -0.0978 0.139

0.312 0.865 0.183 0.0474 0.682 0.558

20 20 20 20 20 20

To-In 0.0162 0.129 0.270 0.428 0.351

0.946 0.587 0.249 0.0599 0.129

20 20 20 20 20

Form-In 0.654 0.273 0.606 0.742

0.00175 0.244 0.00465 0.000182

20 20 20 20

Acet-In 0.121 0.744 0.609

0.612 0.000171 0.00438

20 20 20

Xyl-In 0.398 0.529

0.0824 0.0165

20 20

Temp. 0.715

0.000393

20

Humidity (%)

The pair(s) of variables with positive correlation coefficients and P values below 0.050 tend to increase together. For the pairs with negative correlation coefficients and P values below 0.050, one variable tends to decrease while the other increases. For pairs with P values greater than 0.050, there is no significant relationship between the two variables.

**Paired t-test for inside and outside**

**Paired t-test:** Sunday, September 17, 2017, 12:39:55 AM

**Data source:** Data 1 in Salons 2 (Hopke Suggestions).JNB

**Normality Test (Kolmogorov-Smirnov)** Passed (P = 0.790)

**Treatment Name N Missing Mean Std Dev SEM**

Ben-In 20 0 7.536 1.871 0.418

Ben-Out 20 0 3.669 1.461 0.327

Difference 20 0 3.867 1.232 0.275

t = 14.042 with 19 degrees of freedom.

95 percent two-tailed confidence interval for difference of means: 3.290 to 4.443

Two-tailed P-value = 1.747E-011

The change that occurred with the treatment is greater than would be expected by chance; there is a statistically significant change (P = <0.001)

One-tailed P-value = 8.737E-012

The sample mean of treatment Ben-In exceeds the sample mean of treatment Ben-Out by an amount that is greater than would be expected by chance, rejecting the hypothesis that the population mean of treatment Ben-Out is greater than or equal to the population mean of treatment Ben-In. (P = <0.001)

Power of performed two-tailed test with alpha = 0.050: 1.000

Power of performed one-tailed test with alpha = 0.050: 1.000

**Paired t-test:** Sunday, September 17, 2017, 12:40:18 AM

**Data source:** Data 1 in Salons 2 (Hopke Suggestions).JNB

**Normality Test (Kolmogorov-Smirnov)** Passed (P = 0.142)

**Treatment Name N Missing Mean Std Dev SEM**

To-In 20 0 9.179 3.030 0.678

To-Out 20 0 5.313 1.980 0.443

Difference 20 0 3.865 1.593 0.356

t = 10.848 with 19 degrees of freedom.

95 percent two-tailed confidence interval for difference of means: 3.120 to 4.611

Two-tailed P-value = 0.00000000140

The change that occurred with the treatment is greater than would be expected by chance; there is a statistically significant change (P = <0.001)

One-tailed P-value = 0.000000000698

The sample mean of treatment To-In exceeds the sample mean of treatment To-Out by an amount that is greater than would be expected by chance, rejecting the hypothesis that the population mean of treatment To-Out is greater than or equal to the population mean of treatment To-In. (P = <0.001)

Power of performed two-tailed test with alpha = 0.050: 1.000

Power of performed one-tailed test with alpha = 0.050: 1.000

**Paired t-test:** Sunday, September 17, 2017, 12:40:06 AM

**Data source:** Data 1 in Salons 2 (Hopke Suggestions).JNB

**Normality Test (Kolmogorov-Smirnov)** Passed (P = 0.112)

**Treatment Name N Missing Mean Std Dev SEM**

EB-In 20 0 14.001 4.211 0.942

EB-Out 20 0 6.966 3.040 0.680

Difference 20 0 7.035 2.859 0.639

t = 11.004 with 19 degrees of freedom.

95 percent two-tailed confidence interval for difference of means: 5.697 to 8.373

Two-tailed P-value = 0.00000000110

The change that occurred with the treatment is greater than would be expected by chance; there is a statistically significant change (P = <0.001)

One-tailed P-value = 0.000000000551

The sample mean of treatment EB-In exceeds the sample mean of treatment EB-Out by an amount that is greater than would be expected by chance, rejecting the hypothesis that the population mean of treatment EB-Out is greater than or equal to the population mean of treatment EB-In. (P = <0.001)

Power of performed two-tailed test with alpha = 0.050: 1.000

Power of performed one-tailed test with alpha = 0.050: 1.000

**Paired t-test:** Sunday, September 17, 2017, 12:41:58 AM

**Data source:** Data 1 in Salons 2 (Hopke Suggestions).JNB

**Normality Test (Kolmogorov-Smirnov)** Passed (P = 0.431)

**Treatment Name N Missing Mean Std Dev SEM**

Xyl-In 20 0 18.008 4.171 0.933

Xyl-Out 20 0 7.308 1.853 0.414

Difference 20 0 10.701 2.984 0.667

t = 16.037 with 19 degrees of freedom.

95 percent two-tailed confidence interval for difference of means: 9.304 to 12.097

Two-tailed P-value = 1.688E-012

The change that occurred with the treatment is greater than would be expected by chance; there is a statistically significant change (P = <0.001)

One-tailed P-value = 8.442E-013

The sample mean of treatment Xyl-In exceeds the sample mean of treatment Xyl-Out by an amount that is greater than would be expected by chance, rejecting the hypothesis that the population mean of treatment Xyl-Out is greater than or equal to the population mean of treatment Xyl-In. (P = <0.001)

Power of performed two-tailed test with alpha = 0.050: 1.000

Power of performed one-tailed test with alpha = 0.050: 1.000

**Paired t-test:** Sunday, September 17, 2017, 12:40:34 AM

**Data source:** Data 1 in Salons 2 (Hopke Suggestions).JNB

**Normality Test (Kolmogorov-Smirnov)** Passed (P = 0.657)

**Treatment Name N Missing Mean Std Dev SEM**

Form-In 20 0 11.882 5.885 1.316

Form-Out 20 0 5.642 2.965 0.663

Difference 20 0 6.240 3.355 0.750

t = 8.316 with 19 degrees of freedom.

95 percent two-tailed confidence interval for difference of means: 4.670 to 7.810

Two-tailed P-value = 0.0000000937

The change that occurred with the treatment is greater than would be expected by chance; there is a statistically significant change (P = <0.001)

One-tailed P-value = 0.0000000469

The sample mean of treatment Form-In exceeds the sample mean of treatment Form-Out by an amount that is greater than would be expected by chance, rejecting the hypothesis that the population mean of treatment Form-Out is greater than or equal to the population mean of treatment Form-In. (P = <0.001)

Power of performed two-tailed test with alpha = 0.050: 1.000

Power of performed one-tailed test with alpha = 0.050: 1.000

**Paired t-test:** Sunday, September 17, 2017, 12:41:31 AM

**Data source:** Data 1 in Salons 2 (Hopke Suggestions).JNB

**Normality Test (Kolmogorov-Smirnov)** Passed (P = 0.780)

**Treatment Name N Missing Mean Std Dev SEM**

Acet-In 20 0 32.671 12.592 2.816

Acet-Out 20 0 14.761 5.281 1.181

Difference 20 0 17.910 8.280 1.851

t = 9.674 with 19 degrees of freedom.

95 percent two-tailed confidence interval for difference of means: 14.035 to 21.785

Two-tailed P-value = 0.00000000896

The change that occurred with the treatment is greater than would be expected by chance; there is a statistically significant change (P = <0.001)

One-tailed P-value = 0.00000000448

The sample mean of treatment Acet-In exceeds the sample mean of treatment Acet-Out by an amount that is greater than would be expected by chance, rejecting the hypothesis that the population mean of treatment Acet-Out is greater than or equal to the population mean of treatment Acet-In. (P = <0.001)

Power of performed two-tailed test with alpha = 0.050: 1.000

Power of performed one-tailed test with alpha = 0.050: 1.000

**Effect of hair styling on acetaldehyde concentration**

**Linear Regression** Friday, July 27, 2018, 12:29:12 AM

Data source: Data 1 in Salons 2 (Hopke Suggestions).JNB

Acet-In = 20.477 + (1.891 * Shenyon )

N = 20

R = 0.382 Rsqr = 0.146 Adj Rsqr = 0.0984

Standard Error of Estimate = 11.956

Coefficient Std. Error t P VIF

Constant 20.477 7.451 2.748 0.013

Shenyon 1.891 1.078 1.753 0.097 1.000

Analysis of Variance:

DF SS MS F P

Regression 1 439.460 439.460 3.074 0.097

Residual 18 2572.942 142.941

Total 19 3012.402 158.547

The dependent variable Acet-In can be predicted from a linear combination of the independent variables:

P

Shenyon 0.097

Not all of the independent variables appear necessary (or the multiple linear model may be underspecified).

The following appear to account for the ability to predict Acet-In (P < 0.05): [ None ]

Normality Test (Shapiro-Wilk) Passed (P = 0.495)

Constant Variance Test: Passed (P = 0.792)

Power of performed test with alpha = 0.050: 0.382

The power of the performed test (0.382) is below the desired power of 0.800.

Less than desired power indicates you are less likely to detect a difference when one actually exists. Negative results should be interpreted cautiously.

**References**

LaGrega, M. D., et al., 2010. Hazardous waste management. Waveland Press.
